# LED-Cured Reflection Gratings Stored in an Acrylate-Based Photopolymer

**DOI:** 10.3390/polym11040632

**Published:** 2019-04-06

**Authors:** Manuel G. Ramírez, Daniel Sirvent, Marta Morales-Vidal, Manuel Ortuño, Francisco J. Martínez-Guardiola, Jorge Francés, Inmaculada Pascual

**Affiliations:** 1 Departamento de Óptica, Farmacología y Anatomía, Universidad de Alicante, Apartado de correos 99, Alicante E-03080, Spain; ramirez@ua.es (M.G.R.); danii.sirvent@gmail.com (D.S.); 2 I.U. Física Aplicada a las Ciencias y las Tecnologías Universidad de Alicante, Apartado de correos 99, Alicante E-03080, Spain; marta.morales@ua.es; 3 Departamento de Física, Ingeniería de Sistemas y Teoría de la Señal, Universidad de Alicante, Apartado de correos 99, Alicante E-03080, Spain; mos@ua.es (M.O.); fj.martinez@ua.es (F.J.M.-G.); jfmonllor@ua.es (J.F.)

**Keywords:** holographic reflection gratings, low-toxicity photopolymer, volume holography, LED-curing

## Abstract

The storage of volume holographic reflection gratings in low-toxicity photopolymers represents a challenge at present since they can be used in many important applications such as biosensors and holographic optical elements. In this context, an acrylate-based photopolymer developed in our research group was employed to study the recording of unslanted holographic reflection gratings at high spatial frequencies. The optimal preparation conditions of the photopolymer layers were determinated. The diffraction efficiencies are measured in both recording and curing stage and a comparative study of these values was realized. In addition, a theoretical study using Kogelnik’s coupled wave theory was carried out with the aim of understanding the diffraction efficiency behaviour of both processes. In this work, a maximum diffraction efficiency of 14.1% was reached after a curing process in 150 µm layers at a recording wavelength of 488 nm. This value represents a good result compared to that reported in the literature and opens the way to reflection mode holography research using low-toxicity material.

## 1. Introduction

Holography is an optical technique that allows the storage and reconstruction of three-dimensional objects in recording materials. The holographic gratings are stored through the interference produced by two spatially overlapping coherent light beams, reference and object. Transmission and reflection mode holography are used to carry out the recording process. In transmission mode, the beams impinge on the same side of the recording material, while in the reflection mode, these beams fall on opposite sides, making it possible to obtain high spatial frequencies. The holographic gratings are unslanted when reference and object beams impinge with the same angle with respect to the normal of the layer plane. Otherwise, slanted holographic gratings will be recorded inside the material. 

Many researchers have carried out in transmission mode holography since this recording geometry allows the comparison and characterization of different materials [1,2,3]. Nevertheless, the interest in reflection holographic gratings has increased in recent years due to the demand of materials with enough resolution for high spatial frequencies recording. The reflection holograms have been employed for holography display [2,4], optical data storage, and three-dimensional multiplexing to improve the data storage density [5,6]. One of the main advantages of reflection holograms is that they can be reconstructed using white light. This feature has enabled researchers to focus on the development of sensors for different types of analytes [7,8,9]. Thermosensitive reflection holograms have also been investigated [10]. 

Different types of recording materials have been used in past decades such as dichromate gelatine [11,12], silver halide emulsion [13,14], photopolymers [15,16], photoresist [17,18], and photorefractive [19]. Among them, photopolymer materials presently attract great interest due to their properties, such as high sensitivity, chemical versatility for the design and preparation of different compositions, self-processing capabilities, large dynamic range, good dimensional stability, and relatively low cost. Photopolymers are used in many applications, such as fabrication of holographic optical elements (HOEs) [20,21], holographic sensors [7,22], non-image systems [23,24,25], holographic memories [26], and holographic waveguides [27,28]. Considering the characteristics of photopolymers and consequently their wide variety of applications, a lot of research is being carried out with the aim of developing photopolymer materials with improved properties. One of these properties must be the low-toxicity and environmental compatibility of all the components present in the photopolymer compositions. 

Today’s society demands scientific development based on green technologies to reduce the environmental impact as much as possible. In this sense, the interest in the development of low-toxicity photopolymers for holographic recording in reflection mode has achieved great relevance in recent years. The majority of hydrophilic photopolymers investigated contain poly(vinyl alcohol) (PVA), gelatin binders, or monomers related to acrylamide [16,29,30,31]. The last compound is carcinogenic and toxic when used in its monomer form. Commercial photopolymers by Dupont [32] and Bayer [33] have also been used for holographic recording in reflection mode. Dupont photopolymer causes some problems due to skin contact, while there is no information available about Bayer photopolymer composition and its possible toxicity. In order to avoid this type of risk for health and environment, we employed an acrylate-based photopolymer developed in our researcher group as a recording holographic material for optical applications. This water-soluble holographic photopolymer has low toxicity and good recycling properties [34,35,36,37].

In our previous studies, holographic transmission gratings were stored in an acrylate-based photopolymer at different spatial frequencies of 1205 and 1144 lines/mm for recording laser wavelengths of 488 and 514 nm, respectively [34,36]. The preparation conditions of the prepolymer solutions in that work were controlled to obtain layers with the suitable optical properties that provide the highest diffraction efficiencies (*DE*), i.e., the ratio between the power of diffracted and incident beams was around 90% in 300 µm thick photopolymer layers. Volume phase transmission lenses have also been fabricated in this photopolymer [21]. However, there are no study details about reflection grating stored in this material. This fact represents a challenge and promotes the research realized in this work.

The strict control in the preparation conditions of photopolymer layers acquires greater relevance when high spatial frequencies are stored in the material due to whichever layer imperfection in them could cause decrease in their *DE*. As spatial frequency increase, the *DE* becomes lower as the material cannot correctly resolve the high number of interference fringes. One method to improve the photopolymer response, at high spatial frequency, is to reduce the polymer chain length by adding chain transfer agents (CTA), such as acid citric [38] or 4,4’-azobis(4-cyanopentanoic acid) [39] to the prepolymer solution. Furthermore, free radical scavengers (FRS), such as glycerol [40], can also be incorporated into the composition with the aim of stopping the growth of short polymer chains. A low-toxicity diacetone acrylamide-based material containing citric acid and glycerol has been reported to store a 3050 lines/mm grating in mode reflection with a *DE* of up to 50%. At this point, it is necessary to emphasize that for the correct comparison of *DE* values, the holographic gratings should have similar spatial frequencies. 

In the present work, unslanted holographic reflection gratings were stored in low-toxicity photopolymer at a high spatial frequency of 4738 lines/mm. CTAs and FRSs were not used in this research in order to observe the acrylate-based photopolymer behaviour in reflexion mode (with the same composition as that used in other works in which transmission gratings were stored). A detailed study to optimize the preparation conditions of the photopolymer layers was first carried out. In order to provide temporary stability to the holographic reflection gratings, a curing process was realized. *DE* was measured immediately after both recording and curing processes and its values were compared. With the aim to understand the behaviour of the *DE*, Kogelnik’s coupled wave theory was used to fit the experimental values and to obtain parameters such as the refractive index modulation and the optical thickness. 

## 2. Materials and Methods 

### 2.1. Material Preparation

Holographic reflection gratings were stored in low-toxicity and water-soluble photopolymer (the average refractive index is *n* ~ 1.5). The prepolymer solution was composed of poly(vinyl alcohol) (PVA) as an inert binder polymer, sodium acrylate (NaAO) as a polymerizable monomer, triethanolamine (TEA) as coinitiator and plasticizer, and sodium salt 5′-riboflavin monophosphate (RF) as sensitizer dye. The solvent used was water as all components were soluble. The optimized concentrations in the prepolymer solution were 13.0 w/w %, 0.39 M, 9.0 × 10^−3^ M, 1.0 × 10^−3^ M for PVA, NaAO, TEA, and RF, respectively. All compounds were purchased from Sigma-Aldrich Quimica SL (Madrid, Spain). 

The prepolymer solution was manually deposited over levelled thin and flat glass plates (*n* = 1.5255 at *λ* = 589 nm, 6.3 × 6.3 cm^2^, with a thickness of 0.55 mm, (Marienfeld GmbH & Co. KG, Lauda-Königshofen, Germany) under red light in which the material was not sensitive. The deposited solution was left inside an incubator (Climacell 111, MMM Medcenter Einrichtungen GmbH, Munich, Germany) with controlled conditions (60 ± 5% relative humidity and 20 ± 1° temperature). The photopolymer layers were ready for recording (see results Section 3.2) when enough water had evaporated to reach equilibrium with the environmental conditions inside the incubator. The optimum drying times for these photopolymer layers were studied in this work. The environmental conditions during the recording stage must be rigorously controlled in order to avoid precipitation of NaAO on the surface of the photopolymer layers and therefore obtain reproducible *DE* values. For this reason, environmental conditions at the laboratory must be the same as those used in the drying process.

Hologram stability was ensured and increased using a curing process with an LED lamp (13.5 W, 875 lm at 6500 K, Lexman, Alicante, Spain). The influence of the curing process on *DE* is investigated in Section 3.2. The physical thickness of the photopolymer layers (*d*_h_) was measured with an ultrasonic pulse-echo gauge (PosiTector 200, DeFelsko, Ogdensburg, NY, USA).

### 2.2. Holographic Reflection Setup

**Figure 1:**
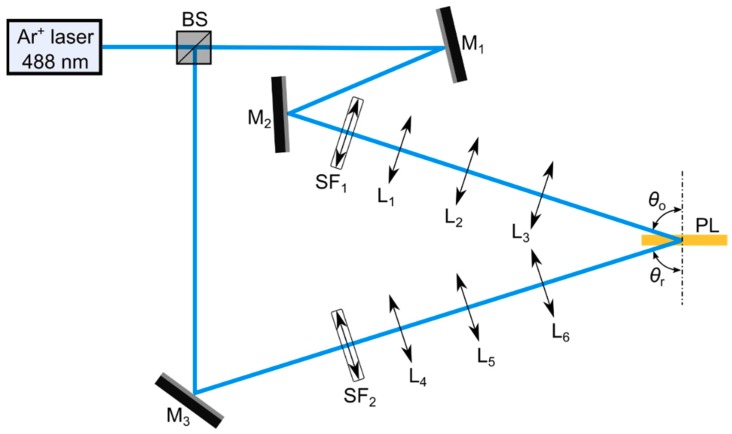
Holographic setup for reflection gratings. BS: beam splitter; SF_i_: spatial filters (microscope objective and pinhole); M_i_: mirrors; L_i_: lenses; *θ_ο_*, *θ_r_*: object and reference recording angle; PL: photopolymer layer.

The experimental holographic setup used is shown in Figure 1. A continuous (CW) Argon Ion Laser BeamLok 2060-10S S/N 944 (Spectra-Physics, Santa Clara, CA, USA) emitting at *λ* = 488 nm (wavelength of the recording light), at which the material is sensitive, was split into two secondary beams, object and reference beams, using a beam splitter (Newport, Irvine, CA, USA). Both beams were spatially filtered and collimated to yield a planewave. The beam diameter was reduced to 0.375 cm using an afocal lens system (L_2_-L_3_, L_5_-L_6_) with the aim of increasing the radiant exposure (*H*) available. The holographic reflection grating was recorded by the appropriate interference between the object and the reference beam. The two laser beams were spatially overlapped at the sample, reaching symmetrically the opposite sides of the photopolymer layer with a recording angles (out of the material) *θ_ο_* = *θ_r_* = 72.9 ± 0.1° with respect to the normal incidence. The exposure times were varied to obtain a range of *H* from 70 to 820 mJ/cm^2^. 

The interference of both beams inside the photopolymer layer results in the formation of bright (constructive interference) and dark (destructive interference) zones. A radical polymerization reaction occurs in the bright zones and a refractive index modulation (Δ*n*) is generated. The mechanism for this reaction has been reported in the literature [41]. When the RF molecules absorb photons at bright zones a singlet excited state (^1^RF*) is produced. After that, a triplet excited state (^3^RF*) is generated by an intersystem crossing. The ^3^RF* reacts with the co-initiator TEA (electron donor), producing TEA radicals which are combined with acrylate monomers to generate chain initiators.

According to Bragg’s law for symmetrical reflection geometry (Equation (1)), the theoretical spatial period (Λ) of the reflection grating was 0.211 µm (equivalent to a frequency of 4738 lines/mm).

(1) $$\Lambda =\frac{\lambda }{2\;\sqrt{n^{2}-sin^{2}\theta }}$$

The gratings obtained in this work are considered as volume hologram gratings because the *Q* factor (Equation (2)) is much higher than 10, particularly between 394 and 951 for the range of effective optical thicknesses (*d*) obtained. 

(2) $$Q=\;\frac{2\pi \lambda d}{n\Lambda^{2}}$$

### 2.3. Analysis of Experimental Data Using Kogelnik’s Coupled Wave Theory

The diffracted beam of a holographic grating recorded in a reflection symmetric setup is overlapped with the Fresnel reflection of the other one. For this reason, it is not possible to evaluate the diffraction efficiency of these gratings as in transmission grating works [36]: by the holographic reconstruction at different angles. The *DE* of the reflection gratings prepared were evaluated by taking advantage of their high spectral selectivity. The Bragg wavelength, at which the maximum *DE* (*DE*_max_) is obtained, is preferentially reflected when the hologram is illuminated with white light. *DE* can be obtained as a function of the photopolymer layer transmittance with (*T*_pg_) and without (*T*_p_) the stored grating (Equation (3)). The transmission spectra at normal incidence upon the photopolymer samples were measured with a double beam spectrophotometer (V-650, Jasco, Madrid, Spain).

(3) $$DE=\frac{T_{p}-T_{\mathrm{pg}}}{T_{p}}$$

Kogelnik’s coupled wave theory [42] provides a useful approach to the parameters that determine the *DE* in sinusoidal volume gratings, considering a phase holographic grating and a null electric conductivity of the photopolymer, (Equation (4)).

(4) $$DE=e^{\left(-\alpha d/\cos\theta^{\prime }\right)}{\left(1+\frac{1-\frac{\xi^{2}}{\nu^{2}}}{\sinh^{2}\sqrt{\nu^{2}-\xi^{2}}}\right)}^{-1}$$

However, as previously mentioned, in our experiment, as it was not possible to measure the diffracted beam, experimental transmission spectra were used to obtain the *DE*. These experimental transmission values as a function of the wavelength can also be fitted by means of efficient transmission (*TE*) relation of Kogelnik’s coupled wave theory (Equation (5)). The parameters *d*, Δ*n*, Λ, *n*, absorption loss coefficient (α), and fringe tip angles (*ϕ*) were obtained, considering a reconstruction angle (inside of the material) *θ*′ = 0.0 ± 0.01°. By calculating the theoretical *TE*, it would also be possible to calculate the theoretical *DE*, although in this work, the last parameter was previously obtained by using the experimental values in Equation (3).

(5) $$TE=e^{\left(-\alpha d/\cos\theta^{\prime }\right)}\left[1-{\left(1+\frac{1-\frac{\xi^{2}}{\nu^{2}}}{\sinh^{2}\sqrt{\nu^{2}-\xi^{2}}}\right)}^{-1}\right]$$

The parameter that controls *DE* at Bragg condition is ν (Equation (6)), while the *ξ* parameter (Equation (7)) is related to the deviation from the exact Bragg condition. As we can observe, *ξ* = 0 maximizes the *DE* value (*DE*_max_).

(6) $$\nu =i\frac{\pi \Delta nd}{\lambda \sqrt{c_{r}\;c_{S}}}$$

(7) $$\xi =-\frac{\pi d}{\Lambda \;c_{S}}\left[\left|\sin\left(\theta^{\prime }-\varphi \right)\right|-\frac{\lambda }{2\;n\;\Lambda }\right]$$

Slant factors (*c_r_* and *c_s_*) are defined in Equations (8) and (9), respectively.

(8) $$c_{r}=\cos\theta^{\prime }$$

(9) $$c_{S}=\cos\theta^{\prime }-\frac{\lambda }{n\;\Lambda }\sin\varphi$$

## 3. Results and Discussion

### 3.1. Optimization of the Preparation Conditions of Photopolymer Layers

**Figure 2:**
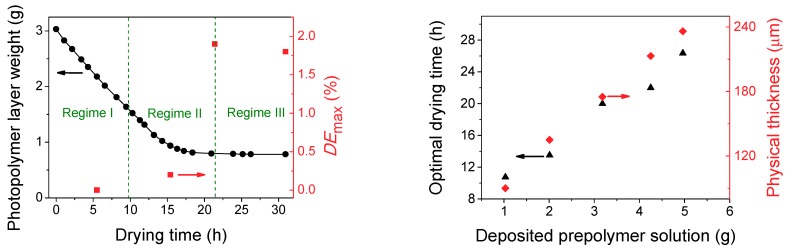
(**a**) Weight loss (black circles) and maximum diffraction efficiencies (*DE*_max_, red squares) of a photopolymer layer when an initial amount of 3.030 g prepolymer solution is deposited over a glass plate. (**b**) Optimal drying time (black triangles) and physical thickness (red diamonds) obtained for different amounts of prepolymer solution deposited. Errors are ±0.001 g in the photopolymer layer weight and deposited prepolymer solution, ±0.017 h in the drying time and optimal drying time, ±1 µm in the physical thickness and ±0.6% in the maximum diffraction efficiencies (*DE*_max_).

The photopolymer layer uniformity was highly sensitive to drying and environmental conditions during the exposure stage. The high concentration of NaAO in the prepolymer solution together with the content of TEA can lead to crystallization during the drying process. TEA has the capacity to form H–bonds with water, therefore a low concentration of TEA makes it more difficult to dissolve NaAO. This fact causes the formation of small crystallization nucleus inside the material giving rise to diffusion processes of light when the photopolymer layers are illuminated with the laser wavelength during the recording stage. In this sense and with the aim to determinate the optimal drying time in which the higher *DE*_max_ is obtained, the weight of the photopolymer layers was monitored as a function of the time that the films remain inside an incubator with controlled humidity (60%) and temperature (20 °C). Figure 2a shows the weight loss of a photopolymer layer when an initial amount of 3.030 ± 0.001 g prepolymer solution is deposited over a glass plate and introduced in the incubator. Three regimens with different drying speeds can be clearly observed. In Regime I, a loss of 47.5% with respect to the initial weight of the photopolymer layer occurs during the first 10 hours of drying. From this time, the drying speed decreases (Regime II). The weight measured decreases very slightly and remains practically constant from 21 hours until the measurement is finished. It is denoted in Figure 2a as regime III. 

In order to obtain the optimal drying time, the *DE*_max_ was measured for four drying times. A total *H* = 600 mJ/cm^2^ was used in the recording stage. The values of *DE*_max_ achieved are also shown in Figure 2a. If the photopolymer layer is illuminated with the recording laser wavelength in Regime I, the diffraction efficiencies achieved will be very close to zero. The higher water content in the layer facilitates the diffusion of polymer chains and the interference fringes will not form. To a lesser extent, a similar effect occurs in Regime II and a small value of *DE*_max_ can be measured. The highest value of *DE*_max_ is obtained when Regime III begins, at 21 h of drying. This time value is selected as the optimal drying time. The *DE*_max_ at 31 h of drying time is slightly lower compared to the highest value of *DE*_max_. This decrease of *DE*_max_ in Regime III is a consequence of possible NaAO precipitation on the surface of the photopolymer layer. Because of this, the environmental conditions during the recording stage must be strictly controlled.

Identical experiments were realized for different amounts of prepolymer solution deposited over the glass plates. The optimal drying time was obtained following the same procedure described for the photopolymer layer in Figure 2a, by finding out the drying time in which the photopolymer layer weight remains practically constant. In addition, the physical thicknesses as a function of the prepolymer solution amount deposited were obtained (Figure 2b). Both optimal drying time and physical thickness show a linear trend with the deposited prepolymer solution in the investigated range. This is very important information in order to know the optimal drying time for a certain photopolymer layer thickness. If we need to store holograms in photopolymer layer with a certain thickness for a particular application, from Figure 2b we can see what the optimum drying time is according to which the recording process should be carried out. It is remarkable to note that this study must be done for each particular composition of the prepolymer solution since the weight loss could be affected by the concentration of the components. 

### 3.2. Influence of Cured Time and Radiant Exposure on DE

The *DE*_max_ values shown in Figure 2a were measured without carrying out a subsequent curing stage after the recording. The holographic gratings are not stable and the *DE* decreases over time. This reduction of *DE* is due to the concentration gradient generated during the recording stage. Molecular diffusion processes are produced inside the photopolymer layers in which the interferential pattern is stored. Diffusion of short polymer chains out of the exposed zones is produced. On the other hand, the monomer and dye molecules that remain in the non-exposed zones diffuse towards exposed zones where the dye concentration is negligible. Therefore, a curing process is necessary to remove the concentration gradient and provide temporal stability to the holographic gratings, which can thereby be used in optical devices for different applications. Several techniques are employed with the aim of increasing the stability of the photopolymer-based holograms. Dehydration of the photopolymer layers under controlled temperature conditions, UV exposure, and incoherent light (LED lamp) are the most used techniques [35,43,44]. The curing process based in a LED lamp is simpler and cheaper compared to those that use UV lasers. Cody et al. carried out UV curing by illuminating diacetone acrylamide-based photopolymer layer with a nanosecond pulsed laser at 355 nm after the recording stage [45]. In that work, the performance of the gradients exposed to UV light remained constant while the layers that did not receive treatment showed a *DE* decrement over time. In the present work and following the same line as our previous papers [35,36], LED exposure post-recording was employed.

**Figure 3:**
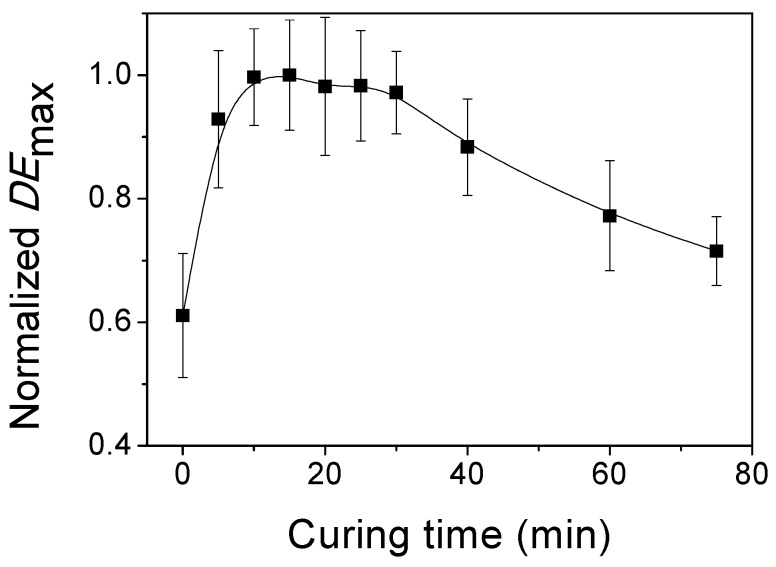
Normalized maximum diffraction efficiencies vs curing time for a photopolymer layer of *d*_h_ = 150 ± 1 µm. *H* = 700 mJ/cm^2^ was used in the recording stage. Error is ±1 min in the curing time.

It is very important to identify the curing times for which the highest *DE*_max_ value are obtained. The normalized *DE*_max_ is represented as a function of the curing time for one holographic reflection grating in Figure 3. The physical thickness of the photopolymer layer was 150 ± 1 µm and a *H* = 700 mJ/cm^2^ was used in the recording stage. The highest *DE*_max_ value was obtained for a curing time range of 11 to 30 min in which *DE*_max_ remains practically constant, while the *DE*_max_ decreases slightly from 30 to 75 min. This *DE*_max_ behaviour after the curing stage is different from that of other photopolymers described in the literature [5]. Generally, the curing process decreases in the *DE*_max_ but, in the present work, the *DE*_max_ increases after the curing stage. In the next section, more details are offered to understand this effect. Finally, a curing time of 20 min was selected for carrying out the curing process of the photopolymer layers after recording stage. 

**Figure 4:**
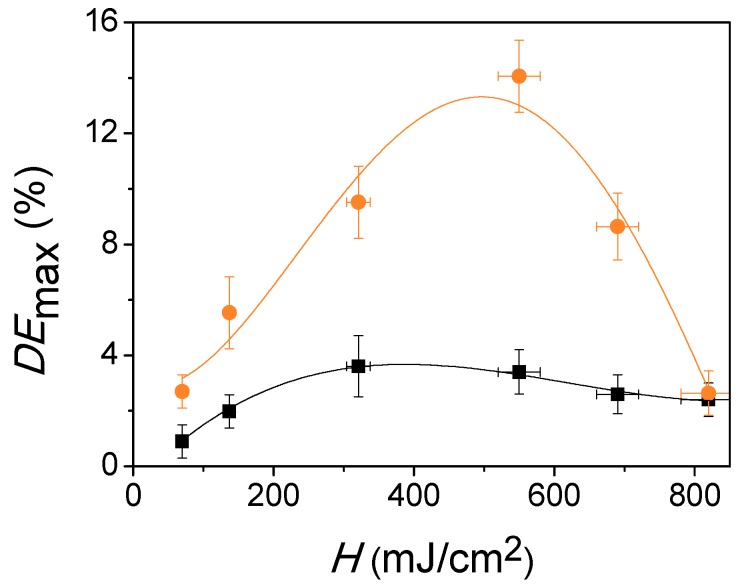
Normalized maximum diffraction efficiencies (*DE*_max_) vs radiant exposure (*H*) for recording (black squares) and curing (orange circles) stages. The lines represent the experimental data polynomial fit.

Once the physical thickness of the photopolymer layers, the optimum drying time (as explained in Section 2.1 and Section 3.1, respectively), and curing time were selected to obtain the highest *DE*_max_ value, the influence of *H* on the *DE*_max_ after both recording and curing stages was investigated. The results are shown in Figure 4. Since the diffracted beam overlaps the reflected beam due to the symmetric geometry employed in the recording stage, the behaviour of the *DE*_max_ as a function of *H* cannot be measured in real time. For this reason, several photopolymer layers with a physical thickness of 150 µm were used for recording reflection gratings with spatial frequencies of 4738 lines/mm. As clearly observed in Figure 4, the *DE*_max_ measured immediately after the recording stage (denoted as *DE*_RS_) grows from 0.9% until reaching a maximum (*DE*_max, RS_) of 3.7% at 400 mJ/cm^2^ (fitted values). Further, *H* increment above this value did not lead to the improvement of *DE*_max_. The explanation of this behaviour is that Δ*n* stored is not enough. The energetic sensitivity, defined as the minimum *H* required to achieve the maximum *DE*, is 400 mJ/cm^2^. This energetic sensitivity is the same magnitude order as those obtained for acrylamide-based reflection mode photopolymer [5,38]. On the range of *H* studied, all *DE*_max_ increase after the curing process (denoted as *DE*_CS_). In Figure 4, it can be observed that the *DE*_CS_ grows from 3.0% until reaching a maximum (*DE*_max*, CS*_) of 13.3% at 495 mJ/cm^2^ (polynomial fitted values). From this *H* value, the *DE*_CS_ decreases. When a *H* of 820mJ/cm^2^ is used, the *DE*_max_ difference after the recording and curing stages is 0.2%. Taking into account that the difference between *DE*_max,CS_ and *DE*_max,RS_ is 9.6%, the optimum *H* to obtain the maximum *DE*_max_ is 495 mJ/cm^2^. 

In order to compare the *DE*_max_ obtained in the present work with the values reported in the literature, it is necessary that the spatial frequencies of the holographic reflection gratings are similar. The *DE* of the holographic gratings stored in the photopolymer layers decrease as the spatial frequency increases [38,46]. The dependence between the recording exposure and the *DE* becomes less pronounced when the spatial frequency grows. The *DE*_max_ behaviour can be explained according to diffusion processes of the short polymer chains that take place from exposed zones to non-exposed regions. This diffusion causes a reduction of the refractive index modulation and therefore a decrease in *DE*. Cody et al. obtained a maximum *DE*_max_ of 15% in a low-toxicity diacetone acrylamide-based photopolymer in reflection geometry at 4750 lines/mm [38], and Mikulchyk et al. reached a 20% *DE*_max_ in a 2700 lines/mm reflection grating recorded on a N-isopropylacrylamide-based photopolymer [10]. In our previous paper, a *DE*_max_ of 9.1% in acrylamide-based photopolymer at 5174 lines/mm was obtained [5]. Taking into account these *DE* values reported in other works, the *DE*_max_ of 14.1% (experimental value) obtained in this paper represents an excellent result. Higher *DE*_max_ values could be achieved by decreasing the spatial frequency and adding CTAs and free radical scavenger in the photopolymer composition as shown in the references [40,47,48]. Hence, more studies are necessary in order to optimize the reflection holograms stored in our acrylate-based photopolymer and improve their performance.

### 3.3. Analysis of the Influence of Cured Time and Radiant Exposure on DE

**Figure 5:**
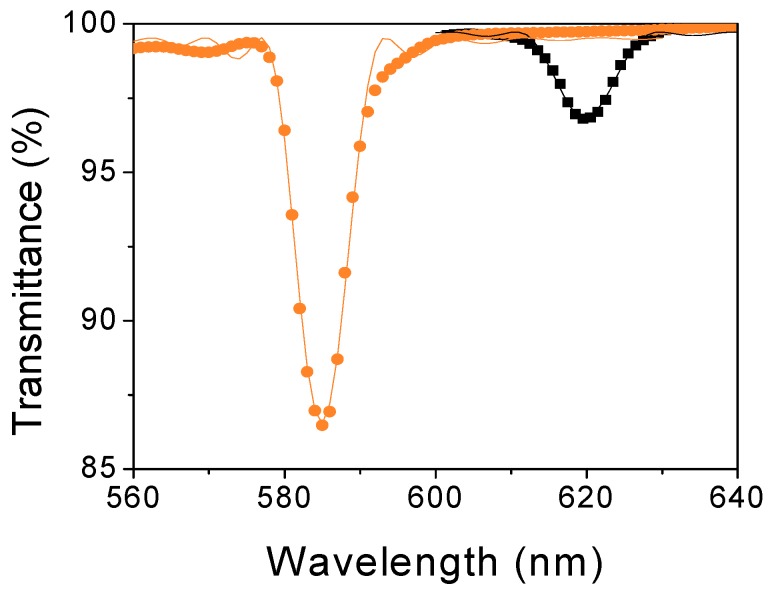
Transmittance of reflection gratings recorded at 550 mJ/cm^2^ for recording (black squares) and curing (orange circles) stages as a function of reconstruction wavelength. The spatial frequency of the gratings was 4738 lines/mm and *d*_h_ = 150 µm. Solid lines represent the theoretical fit through Kogelnik’s coupled wave theory. Errors are ±0.3% in the transmittance and ±1 nm in the wavelength.

In order to understand the *DE*_max_ behaviour between both recording and curing stages, a theoretical analysis of the holographic grating parameters were realized. As explained in Section 2.3, the optical parameters were obtained through the theoretical fit of the experimental data with Kogelnik’s coupled wave theory (Equation (5)). Figure 5 shows the experimental transmittance spectra measured (as percentage) immediately after recording (RS) (black squares) and curing stage (CS) (orange circles) for a reflection grating recorded with a total *H* = 550 mJ/cm^2^. The *DE*_max_ is calculated from the experimental data by means of Equation (3) and the theoretical fits are represented with solid lines. The same procedure was carried out for the rest of reflection gratings represented in Figure 4. A summary of the results is presented in Table 1. 

The *n* values obtained through theoretical fit are between 1.499 and 1.509. For all photopolymer layers, the *ϕ* are close to 90°, which gives an idea of the goodness of fit. The *α* values are lower than 10^−3^·µm^−1^ in all cases. These parameters do not change significantly by the curing process. However, the Δ*n* is one of the most important optical parameters in the *DE* of a holographic grating. The Δ*n* values for both recording (Δ*n*_RS_) and curing (Δ*n*_CS_) stages can be observed in Table 1. Δ*n*_RS_ are smaller than Δ*n*_CS_ in the *H* range investigated. The maximum value of Δ*n*_RS_ is 0.030 at 690 mJ/cm^2^, while the maximum Δ*n*_CS_ value achieved is 0.050 at 550 mJ/cm^2^. For high *H* values from the exposure of 550 mJ/cm^2^ the decrease of Δ*n*_CS_ is more noticeable than Δ*n*_RS_.

Regarding optical thickness, not large variations in this parameter occur when they are compared at the same stage (*d*_RS_ and *d*_CS_ denoted the optical thickness obtained after the recording and curing stage, respectively). These optical thicknesses are smaller than that obtained on stored transmission holograms in photopolymer layers with similar composition [36]. This can be qualitatively explained taking into account the different recording geometries used [39]. When interference fringes are stored by means of reflection geometry, recording beams come from both sides of the sample. The grating is formed from the centre of the photopolymer layer outwards. Theoretically, both beams must be subjected to the same material attenuation, and thus, the interferences would take place correctly. However, a gradient concentration of dye molecules between both sides of the sample could exist, causing an unequal attenuation between them. This fact means that the interferences could not be adequate. On the other hand, the recording beam approach in transmission geometry is from the same side of the sample, and therefore, its attenuation inside the material is similar.

When the curing stage is carried out, both *d*_CS_ and Λ_CS_ decrease with respect to *d*_RS_ and Λ_RS_ values, respectively (Λ_RS_ and Λ_CS_ are the spatial periods obtained after the recording and curing stage, respectively). This fact can be explained due to a dimensional change in the material that can occur during the curing process. The total conversion of the monomer molecules into a polymer network causes a volume reduction produced by the close packing between polymer chains. This is known as photopolymerization shrinkage. It is interesting to note that this phenomenon is very important in the case of reflection gratings due to the high number of interference fringes. From Equation (4), the effect of Δ*n*, *d*, and Λ can be analyzed. Obviously, an increase of *DE* is produced when Δ*n* grows. However, the *DE* decreases when *d* and Λ are lower. The variations in *DE* values when these parameters are modified are not of the same order of magnitude. Although the *DE* decreases due to the variations of *d* and Λ, the greater contribution of Δ*n* causes the final increase of *DE* after the curing process. In this sense, more studies are necessary to modify the composition of the material by adding other components which produce higher Δ*n* values [49,50].

**Figure 6:**
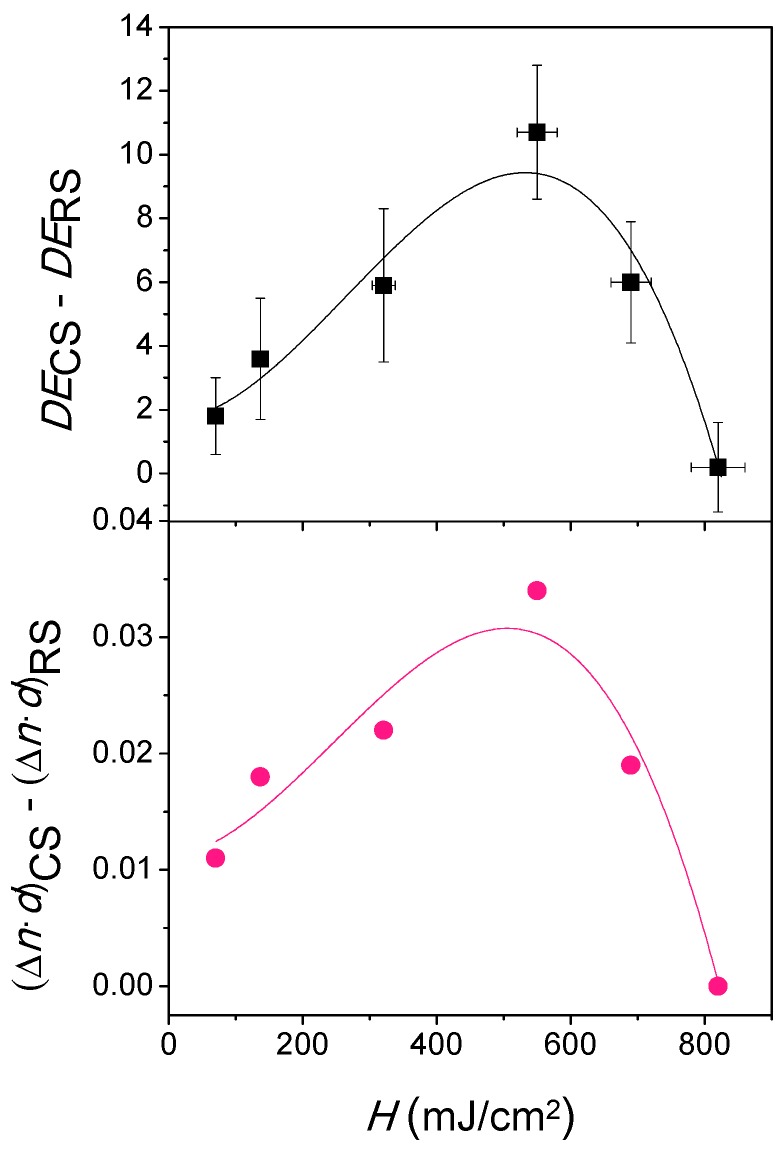
Differences between both curing and recording stage for diffraction efficiencies (*DE*) and Δ*n*·*d* as function as radiant exposure (*H*). The lines represent the experimental data polynomial fit.

From Equation (4), the *DE*_max_ is obtained at the Bragg condition (in which the parameter *ξ* is 0). From this point, *DE* is dominated by parameter *ν*, which depends on the product Δ*n*·*d*. Therefore, and with the aim of understanding the *DE* behaviour between both recording and curing stage, the differences *DE*_CS_–*DE*_RS_ and (Δ*n*·*d*)_CS_–(Δ*n*·*d*)_RS,_ are obtained and represented as a function of *H* in Figure 6. The same trend is clearly observed in both values. The maximum (*Δn*·*d*)_CS_–(*Δn*·*d*)_RS_ difference is reached in the range from 490 to 500 mJ/cm^2^ and it matches with the maximum value of *DE*_CS_–*DE*_RS_. From this maximum, the lowering of *DE*_CS_–*DE*_RS_ is explained by the fact that the Δ*n*·*d* values decrease in both stages although in a different order. As observed in Table 1, (*Δn*·*d*)_CS_ lowers from 0.070 µm at *H* = 550 mJ/cm^2^ until 0.029 µm at *H* = 820 mJ/cm^2^. In the same *H* range, the decreasing of (*Δn*·*d*)_RS_ is less noticeable compared to that which occurs in the curing stage; (*Δn*·*d*)_RS_ is 0.036 µm at *H* = 550 mJ/cm^2^, reaching the same value when *H* is 820 mJ/cm^2^. This trend explains the *DE*_max_ behaviour for both stages observed in Figure 4. 

**Figure 7:**
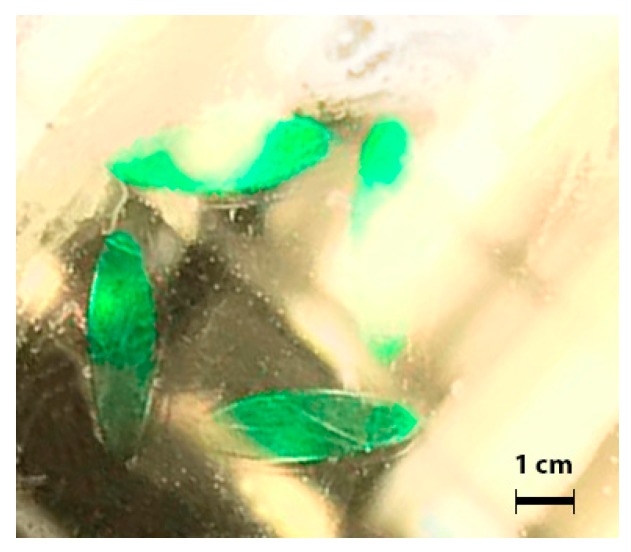
Photography of four holographic reflection gratings daylight illuminated.

A reflection holographic grating inside the photopolymer sample after the curing process is shown in Figure 7. The green areas correspond to the real colour observed by the multilayer optic effect of the grating fringes. The unexposed zones were practically transparent to the sunlight.

## 4. Conclusions

In this paper, reflection holographic gratings of 4738 lines/mm have been presented in a low-toxicity and water-soluble photopolymer, prepared at optimum conditions. A curing process with a low-cost LED lamp that allows the hologram to stabilize over time, as well as increasing the *DE*_RS_ by around 10% with respect to the *DE*_CS_, has been demonstrated. The *DE*_max_ obtained in this work is one of the best values published in reflection holographic gratings over green photopolymers. Considering the dependence of *DE*, discussed in this work, with the spatial frequency, the refractive index modulation, or the length of the polymer chains, we can conclude that higher *DE* will be possible to obtain in the low toxicity material, Biophotopol. In future works, the photopolymer composition will be modified by adding other components in order to increase the refractive index modulation and therefore the *DE*_max_.

## Figures and Tables

**Table 1:** Parameters obtained from theoretical fit of the experimental transmittance values.

*H* (mJ/cm^2^)	Cured	Δ*n*	*d* (µm)	Δ*n*·*d* (µm)	Λ (µm)	*DE*_max_ (%)
70	No (RS *)	0.0012	16.0	0.019	0.207	0.9
	Yes (CS ^¤^)	0.0035	8.6	0.030	0.194	2.7
137	No (RS)	0.0017	16.4	0.028	0.204	1.9
	Yes (CS)	0.0044	10.2	0.046	0.197	5.5
321	No (RS)	0.0018	20.7	0.037	0.207	3.6
	Yes (CS)	0.0036	16.5	0.059	0.198	9.5
550	No (RS)	0.0025	14.4	0.036	0.206	3.4
	Yes (CS)	0.0050	13.9	0.070	0.196	14.1
690	No (RS)	0.0030	10.7	0.032	0.203	2.6
	Yes (CS)	0.0050	10.3	0.051	0.191	8.6
820	No (RS)	0.0027	10.6	0.029	0.201	2.4
	Yes (CS)	0.0030	9.6	0.029	0.192	2.6

* Recording stage, ^¤^ Curing stage.
